# The route to financial sustainability of a large Public Private Partnership—the example of the clinical trial network conect4children (c4c)

**DOI:** 10.3389/fmed.2025.1599997

**Published:** 2025-07-24

**Authors:** Laetitia Paumard, Heidrun Hildebrand, Marianne Engel, Pirkko Lepola, Esther Montero, Frank Tennigkeit, Magda Rosenmöller

**Affiliations:** ^1^IESE Business School, Barcelona, Spain; ^2^Bayer AG, Research and Development Pharmaceuticals, Pediatric Medicine, Berlin, Germany; ^3^Helsinki University Hospital, University of Helsinki, Helsinki, Finland; ^4^Servizo Galego de Saúde (SERGAS), Santiago de Compostela, Spain; ^5^UCB BioSciences, Monheim, Germany

**Keywords:** financial sustainability strategy, Public Private Partnerships, paediatric clinical trials, clinical trial network, conect4children, business school

## Abstract

**Background:**

The financial sustainability of publicly funded initiatives, particularly Public Private Partnerships (PPPs), is crucial for ensuring the intended long-term impact beyond the project’s funding phase. Despite many initiatives aspiring to lasting impact, very few practical examples and little evidence are available on successful financial sustainability strategies, and even fewer are available on the path to achieve the financial autonomy of any successor organisation. This paper intends to shed light on this aspect by exploring the strategy and challenges towards the financial sustainability of conect4children (c4c), an Innovative Medicines Initiative (IMI)-funded PPP establishing a pan-European paediatric clinical trial network to facilitate and support the planning and conduct of paediatric clinical trials.

**Methods:**

The c4c project was planned for a lasting impact from the start. An entire work package dedicated to financial sustainability was already sketched in the proposal and started from the onset of the project, including benchmarking against clinical trial networks globally, needs assessment leading to a value proposition, service portfolio definition, analysis of legal requirements, and the development of a sound business concept.

**Results:**

c4c adopted a combined approach, translating research findings into market-tested services through the parallel set-up of a standalone entity, c4c-Stichting (c4c-s). This strategy offers valuable insights into planning financial sustainability for similar large-scale initiatives and, more broadly, provides a significant contribution to the discourse on financial sustainability. These insights are highly relevant to Regulatory Science, as the sustainability of such infrastructures is critical to the success of regulatory science strategy and long-term public health outcomes.

## 1 Introduction

EU funding instruments such as the Innovative Medicine Initiative (IMI), currently known as the Innovative Health Initiative (IHI),^1^ support promising projects for several years in Europe, aiming to “*translate health research and innovation into tangible benefits for patients and society”*.^2^ Often, once EU funding ends, many of those successful initiatives struggle to ensure a lasting impact beyond the one achieved during the project duration. While there is general acknowledgement of the importance of financial and organisational sustainability, there is little information available about the insights developed and the tools and approaches applied to achieve these goals (1). The realisation of financial sustainability often presents an insurmountable challenge, even for the most promising EU-funded projects. Indeed, despite the number of innovative co-funded Public Private Partnership (PPP) initiatives supported, especially in healthcare (2), there is a lack of a clear understanding, let alone a definition of financial sustainability or hands-on toolkits on how to achieve it. With a limited number of publications on this topic (3), the European Commission’s co-funded EU projects face substantial challenges in bringing their innovative and promising solutions into real and competitive organisational and business models under complex and challenging markets and contexts.

conect4children (c4c), an IMI2-funded 7-year project, was launched in 2018, with financial sustainability as a leading idea and a central piece in the project focus and architecture from the beginning. This paper provides practical and methodological insights on the c4c route to sustainability, including the key steps of developing the financial sustainability process for a PPP, relying on the learnings from the conect4children (c4c) project experience.

### 1.1 Worth being sustainable—the conect4children network

**Figure 1:**
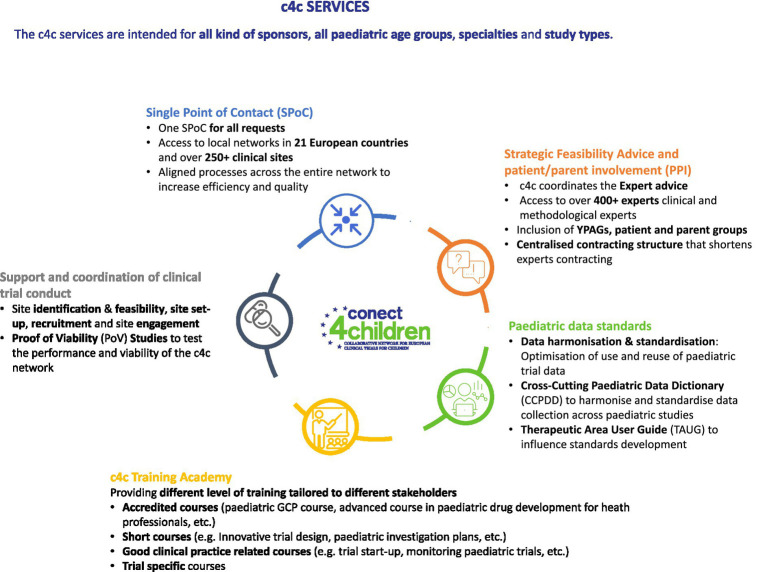
c4c project cutting-edge service offering.

Launched in May 2018, conect4children (c4c),^3^ is an IMI2-funded project,^4^ aiming to create a sustainable, integrated pan-European collaborative paediatric clinical trial network to accelerate, support and facilitate the conduct of clinical trials in children to further contribute to the development of better medicines for babies, children, and young people (4). During the 7-year duration of the project, c4c focused on building the capacity and capability to support multinational paediatric clinical trials for all disease areas and all trial phases—serving all kind of sponsors, all paediatric age groups, specialties, and study types. To do so, c4c developed a set of cutting-edge services (5) (Figure 1) and demonstrated successful proof of concept in optimising paediatric trial facilitation (6) to accelerate the development of new paediatric therapeutics (7).

### 1.2 The structure and aims of the c4c consortium

The c4c consortium includes a wide range of partners across Europe from both academia and industry, bringing together different perspectives and covering diverse needs and experiences in paediatric clinical trials. In detail, conect4children is a one-of-a-kind collaboration between 36 academic partners (including national and specialty networks and paediatric hospitals), 10 industry partners affiliated to the European Federation of Pharmaceutical Industries and Associations (EFPIA), and 500 affiliated partners. Like all IMI projects, c4c is led by both academic and industry partners, both of whom are actively involved in paediatric clinical trials aiming to develop new medicines for children, either as sponsors of trials and users of the network, or as academic clinical trial sites conducting such studies with the support of an extensive network of National Hubs (NHs). During the project, valuable data and information were collected about the network functions, in terms of effectiveness and efficiency, providing learnings taken into the c4c successor organisation, c4c-Stichting (c4c-S).

## 2 Methods—key steps towards financial sustainability

From the project onset, the c4c financial sustainability strategy was aimed at conceiving a business concept to ensure a sustainable network beyond the project funding. Therefore, the aim for financial sustainability guided the transversal work of one of the project’s work packages, Work Package 3 (WP3), focusing on “*Business plan development, expansion of the network and sustainability of the network funding sources post-IMI support*.” Under the combined leadership of both industry and academic partners and in collaboration with all relevant consortium partners in this workstream of the project, WP3 reached broad internal alignment, to build while a shared vision for the c4c successor organisation. Additionally, WP3 benefited from the support of a top-ranked European Business School, IESE^5^, and a law firm experienced in advising EU funded projects, Lawrencium Legal.^6^

**Figure 2:**
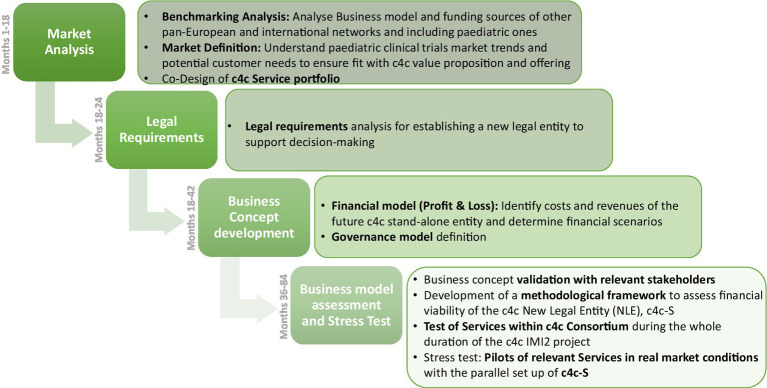
Key steps of c4c route to sustainability.

The different activities of the c4c route to financial sustainability covered four main steps: market analysis, legal requirements, business concept development, and assessment and stress test (Figure 2).

### 2.1 Market analysis and service portfolio definition

The c4c project financial sustainability strategy initially focused on the customer’s needs assessment, benchmarking analysis and market overview to gain an understanding of the following elements:

- the trends of the paediatric clinical trial market in Europe and globally
- benchmarking of existing paediatric clinical research networks (analysis of their business concepts and funding sources through research, surveys, and semi-directed interviews with the networks).
- understanding of customer needs (via customer need surveys with relevant stakeholders)
- defining the c4c value proposition and designing c4c cutting-edge service offerings in alignment with the findings of prior analyses using the Business Model Canvas^7^ framework (8), interactive multi-stakeholders’ workshops and interviews with key categories of stakeholders involved in paediatric clinical trials (including non-c4c-related companies and Clinical Research Organisations (CROs).

### 2.2 Legal considerations

Legal considerations were central to build c4c long-term sustainability strategy, both at the project level—in fostering effective collaboration among large pharmaceutical members within the consortium and in ensuring the optimal delivery of trial-related services—and in the establishment of the new legal entity.

#### 2.2.1 Management of data privacy during the IHI project and beyond

Data privacy and legal considerations are critical in PPPs, especially when large pharmaceutical companies are both members and users of the consortium’s services. For instance, c4c trial related services (site identification, feasibility, expert advice), imply to disclose trial confidential information. As usual in this sector, all clinical data remains the property of the trial sponsors and is strictly protected by robust legal agreements between the sponsor and c4c fostering trust and supporting the long-term sustainability of the network services. Only anonymised, sponsor’s voluntarily shared data was used to assess c4c network service performance. This legal framework—essential for all categories of sponsors—has been fully adopted by the network’s new legal entity.

#### 2.2.2 Legal requirements for establishing a new legal entity

As part of developing the legal strategy for setting up the new legal entity, the initial focus was to identify the most appropriate organisational and legal requirements that would meet the network’s needs—being able to work with academia and industry and generate revenues to fund operations. At the same time, it was essential to ensure full compliance with the IMI requirements for a non-profit entity.

To this end, the legal and regulatory contexts of several countries were assessed to determine the most suitable legal structure, operational design, and corresponding legal framework for the new entity. For instance, the European Research Infrastructure Consortium (ERIC) form has limitations in working with commercial sponsors; therefore, it was not an optimal format for the c4c new legal entity, which aims to provide services to both academic and industry sponsors. Similarly, a for-profit legal structure was not compatible with IMI/IHI requirements for a non-for-profit organisation.

To determine the most appropriate country establishing and operating the c4c successor organisation as a new legal entity, an evaluation of national local legal requirements was performed, resulting in a list of conducted. Based on this legal analysis, three countries were shortlisted as potential hosts for the c4c successor organisation. These were subsequently compared through a SWOT analysis using the following criteria:

- **Overview of different legal forms:** Inventory and comparison of the most relevant legal forms for the c4c successor organisation considering the requirements for establishment, needed capital, transactional costs, time to establish, labour legislation and salaries affecting the overall cost structure for comparison across the pre-selected countries.
- **Taxation:** Evaluation of the taxation’s impact for the c4c successor organisation in the potential host country, taking into consideration taxes associated with both a legal form and its activities. The country tax regime analysis also included the scanning of potential deductions applied on Research & Development (R&D) activities as well as deductions for employees hired as expats.
- **Funding**: Scanning of the funding opportunity landscape in pre-selected countries from both public (especially country- or regional-related support for infrastructure development and the start-up phase) and private sources (charity, philanthropy, etc.).
- **Host country landscape:** Assessment of the attractiveness of the host country environment considering support provided by governmental instances to newcomers to the country, including quality of administrative, financial, and legal services or the level of complexity/flexibility of the country’s administrative and governmental procedures.
- **Headquarters location**: For the pre-selected countries, practical and operational criteria were checked: salary levels, cost of living, transportation infrastructure, attractiveness for talent—its ability to develop and retain talent and an available pool of skilled professionals for key functions at the c4c headquarters. The Netherlands was chosen as the optimal location for c4c headquarters, especially for their facilities, and proximity with EU institutions (Brussels) and regulatory bodies such as the European Medicines Agency (Amsterdam), considered important for the visibility of the successor organisation, along with good availability of practical support by government helpdesks for new organisations.

Based on the scoring results, several legal scenarios were developed to provide a comprehensive overview of the most relevant legal and operational forms for the successor organisation. The c4c consortium jointly opted for a Dutch non-for-profit foundation, *Stichting*—a legal set-up also preferred by previous IMI projects such as Get Real^8^ or EUPATI,^9^ and even by international capital markets (9). Thus, the new legal entity was to be based in the Netherlands, the country favoured in view of the selected legal form, offering attractive conditions for the headquarters and enabling flexible interactions with the c4c National Hubs (NHs) Network, with hubs located in various European countries, under different organisational set-ups and local legal conditions.

### 2.3 Business concept development

Building on insights from benchmarking, needs and market analysis and service portfolio, the business concept development started as early as the second year of the project, with a collective and iterative approach in parallel with the other project tasks, covering governance, operational and financial aspects.

#### 2.3.1 Governance

**Figure 3:**
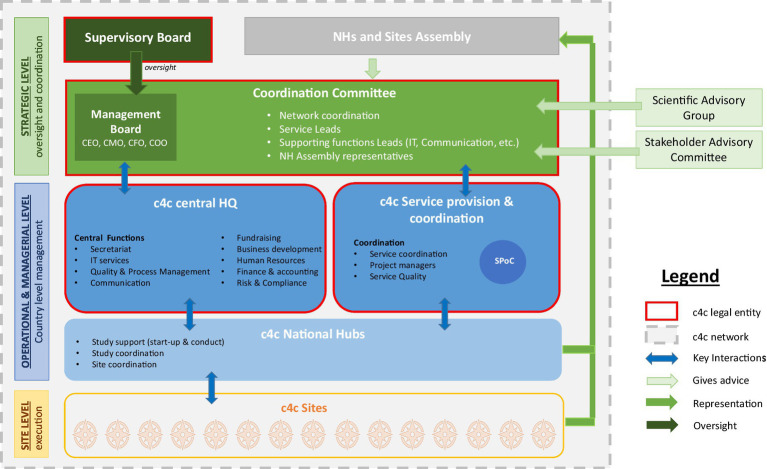
c4c network Governance model that served as the foundation for c4c-S.

The governance and management structure of the network are critical for fostering good practices in all activities and services. The key components of c4c governance are listed below:

- a lean central organisation, reflecting the pan-European network vision, whereas the National Hubs Assembly allows for close collaboration with sites and local key partners.
- good operational and managerial dispositions at the central and decentral levels enable the smooth provision of different services in close relationships with different sites.
- the proposed overall network organisational charter (Figure 3) relies on participative governance, enabling the contribution of all relevant stakeholders, reflecting c4c’s main aim to overcome the fragmentation of clinical research in paediatrics.

#### 2.3.2 Financial model

Early in the project, a detailed financial projection for different scenarios (5 years forecast Profit & Loss) was elaborated, including assumptions for costs and capacities at both the central and country levels (National Hubs). Based on feedback from c4c service leads and partners and first financial projections of revenues, this forecast was completed with three financial scenarios—a base scenario, an optimistic scenario, and a pessimistic scenario.

### 2.4 Business model assessment and stress test

Drawing on the consortium’s experience in delivering c4c services, the business concept and network capabilities were assessed in collaboration with relevant stakeholders. Interviews and co-creation workshops with service providers and potential future customers (including mid-size and large pharmaceutical companies, small biotech firms, SMEs, etc.) allowed to assess customer needs and ensure the fit with corresponding c4c services, and more specifically:

(a) most pressing needs to support the design and conduct of paediatric clinical trials.
(b) the preferred model of interaction with c4c (i.e., membership or fee for service approach, etc.)
(c) the evaluation of the expected demand across the different c4c services.
(d) the identification of potential c4c service portfolio gaps or operational challenges to address.

These customers centred co-creation activities provided valuable insights and feedback on the c4c business concept. Additionally, they serve as an effective means to inform potential future customers about the c4c network and its service offerings.

#### 2.4.1 Methodological framework

An initial financial model was developed as a base for a methodological framework that served as *a “toolkit”* to continuously assess the financial viability of the c4c successor organisation and its service offerings, enabling to adapt it to the fast-changing dynamic healthcare sector.

The c4c project relied on a collective and iterative approach, aiming to design the most suitable business concept for the c4c successor organisation, c4c-Stichting (c4c-S), ensuring that during its start-up phase and beyond, the c4c-S team can rely on this toolkit developed during the project and further adapt it for its convenience.

#### 2.4.2 Business concept stress test

The follow-up organisation, c4c-Stichting (c4c-S), was created in parallel with the last years of the IMI2-funded project, starting in the fifth year, out of 7 years of total c4c project duration. This allowed to test the network capabilities in real market conditions and the business concept suitability in real-life settings. Small pilot evaluations of relevant services provided an opportunity to gather key learnings, enabling early adjustments for network management, service offerings and processes.

## 3 Results—along the road to financial sustainability

### 3.1 Key components of financial sustainability

The following components were identified to be critical for the c4c project and c4c-S financial and organisational sustainability:

- ***Early planning of financial sustainability beyond EU funding:*** From the planning phase, as early as 2016, the financial sustainability strategy was already included in the c4c proposal, building on the detailed recommendations from the IMI Call 10 (Topic 4)^10^ on the creation of a pan-European paediatric clinical trials network.
- ***Key stakeholders’ engagement:*** The c4c project benefited from a broad and engaged set of key stakeholders, whose early involvement was decisive in sustaining initiative in the long term. A year before the project’s launch in April 2017, a workshop was organised to raise awareness and align stakeholders on the policy front, complementing the project’s scientific objectives. This effort included engaging stakeholders in paediatric clinical trials and existing academic and specialty networks to further raise awareness, foster acceptance and potential collaboration with the c4c project.
- ***Financial sustainability as a pivotal component of the project:*** Thoughtful project design combined with strong project leadership ensures sustainability as a pivotal component that plays a central role in project implementation. A full transversal Work Package was dedicated to the development of a sustainability strategy, enabling early implementation of a shared vision and an approach for both the c4c project consortium and its successor, the c4c-Stichting (c4c-S) organisation.
- ***A combined approach:*** In addition, the launch of the successor organisation (c4c-S) in July 2023, at an advanced stage of the c4c IMI2 project, enabled the creation of valuable synergies with the ongoing project, including the testing of the network services (pilot services) in real market conditions, and facilitating knowledge and experience transfer from the project to c4c-S.

### 3.2 Main challenges for financial sustainability

The c4c route to financial sustainability faced several challenges, which were addressed in a variety of ways:

- **Setting-up a long-term vision for the c4c network beyond IMI funding** implied the necessity and complexity of using a mixed approach: simultaneously implementing the IMI2 project and planning the start of the successor organisation, right from the beginning. The mixed approach involves aligning project consortium research activities with a customer-oriented mindset for the creation of a future standalone legal entity.
- **Complex decision-making:** in a PPP, the involvement of multiple stakeholders often involves complex and time-consuming decision-making processes. To address this, the c4c project was designed around lean central coordination and a well-defined participative governance structure that facilitated the information sharing and decision-making process within a large consortium.
- **Different needs and interests from industry and academia:** the involvement of partners with different needs and interests makes alignment with a common financial sustainability strategic approach challenging. All activities were co-led by industry and academia partners to ensure the voice of both perspectives.
- **Public perception:** there might be scepticism or concerns regarding c4c-S, especially from academic sponsors regarding industry interests, as the latter was co-leading the c4c project. However, with the joint work on shared project goals by both industry and academia, the provided expertise, insights and experience will contribute to its success.

## 4 Discussion—Replicability of the c4c financial and organisational sustainability strategy

Despite the c4c IMI2 project specificities, the financial sustainability strategy implemented during the project contains key components that are generalisable and replicable for other initiatives aiming to achieve business continuity beyond project funds. While the c4c project focused specifically on building a network infrastructure within a paediatric setting and further establishing a new legal entity, other initiatives may not follow this exact model, as financial and organisational sustainability can also evolve around scientific findings. Despite the final aim of an initiative, this paper emphasizes the importance of the financial sustainability process and the key elements that are valuable for its development, advocating for an early start with a clear transversal workstream dedicated to the development of a sound business concept and financial sustainability strategy starting from the project’s inception.

Notably, not all IMI/IHI projects intend or need to transition into standalone legal entities; however, for all, the focus should be on the sustainability approach throughout the project. For various EU Commission-funded initiatives considering the establishment of a standalone organisation, it is crucial to develop distinct financial sustainability strategies that differentiate between project results intended for transfer to a successor organisation (including monetisation strategies) and those contributing to research and public knowledge. This paper specifically addresses the design and implementation of the c4c financial and organisational sustainability strategy and highlights critical steps of business concept development during the IMI2 project and even before the project launch, at the proposal stage.

## 5 Conclusions—the importance of strong foundations

**Figure 4:**
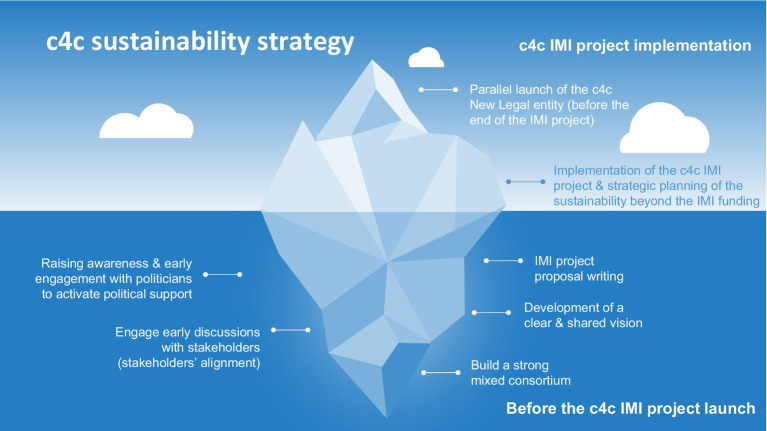
c4c sustainability strategy - building strong foundations.

The design of a financial sustainability strategy prior to the launch of the c4c project (Figure 4) played a crucial role in planning the success of the successor organisation c4c-S. Drawing on insights gained from previous industry and publicly funded initiatives at both the European and international levels, the c4c industry and academic partners developed a clear and shared vision early, identifying the key customer needs and potential pitfalls to build a successful financial and organisational sustainability approach. Learnings from existing and past models, as well as key stakeholder experience in the paediatric clinical trials field, were critical to shaping the c4c project vision. Indeed, a key factor in the project’s success was the consistent shared vision of c4c from its inception and throughout its development across the involved stakeholders.

## Data Availability

The original contributions presented in the study are included in the article/supplementary material, further inquiries can be directed to the corresponding author.

## Footnotes

^1^IMI, now IHI: Innovative Health Initiative: www.ihi.eropea.eu.

^2^“*At IHI, our core goals are to translate health research and innovation into tangible benefits for patients and society, and ensure that Europe remains at the cutting edge of interdisciplinary, sustainable, patient-centric health research*” Mission and objectives | IHI Innovative Health Initiative (europa.eu).

^3^ https://conect4children.org/

^4^IMI Grant agreement number 777389.

^5^ http://www.iese.edu/crhim

^6^ https://lawrencium-legal.com/

^7^The Business Model Canvas is a strategic management and entrepreneurial tool. That allows to describe, design, challenge, invent, and pivot the business model, a very useful method, applied in leading organisations and start-ups worldwide. www.strategyzer.com/library/the-business-model-canvas

^8^ https://getreal-institute.org/

^9^ https://eupati.eu/

^10^IMI Topic 4: Creation of a pan-European paediatric clinical trials network (topic code: IMI2-2016-10-04).

## Glossary

c4c: conect4children
c4c-S: conect4children-Stichting
CCPDD: Cross-Cutting Paediatric Data Dictionary
CROs: Clinical Research Organisations
EFPIA: European Federation of Pharmaceutical Industries and Associations
EMA: European Medicines Agency
ERIC: European Research Infrastructure Consortium
GCP: Good Clinical Practices
IHI: Innovative Health Initiative
IMI: Innovative Medicine Initiative
NHs: National Hubs
NLE: New Legal Entity
P&L: Profit and Loss
PoV: Proof of Viability
PPI: Patient Parent Involvement
PPPs: Public Private Partnerships
R&D: Research and Development
SPoC: Single Point of Contact
SWOT: Strengths, Weaknesses, Opportunities, Threats
TAUG: Therapeutic Area User Guide
WP: Work Package
YPAGs: Young People’s Advisory Groups
